# Phenolic Profile, Antioxidant Capacity, and Alpha-Glucosidase Inhibitory Activity of High-Oil Corn Doubled-Haploid Hybrids from Mexico

**DOI:** 10.3390/molecules31101654

**Published:** 2026-05-14

**Authors:** Cynthia A. López-Torres, Nancy Y. Salazar-Salas, Gabriela López-Angulo, Francisco Delgado-Vargas, Karen V. Pineda-Hidalgo, Alicia Navarro-Leyva, Ricardo E. Preciado-Ortíz, Luis A. Peinado-Fuentes, José A. López-Valenzuela

**Affiliations:** 1 Posgrado en Ciencia y Tecnología de Alimentos, Facultad de Ciencias Químico-Biológicas, Universidad Autónoma de Sinaloa, Av. Josefa Ortiz y Av. Américas S/N, Culiacán C.P. 80013, Sinaloa, Mexico; cynthia.lopez.torres@gmail.com (C.A.L.-T.); nancy.salazar@uas.edu.mx (N.Y.S.-S.); fdelgado@uas.edu.mx (F.D.-V.); kvpineda@uas.edu.mx (K.V.P.-H.); alicia.navarro@uas.edu.mx (A.N.-L.); 2 Campo Experimental Bajío, Instituto Nacional de Investigaciones Forestales, Agrícolas y Pecuarias, Km. 6.5 Carretera Celaya-San Miguel de Allende, Celaya C.P. 38110, Guanajuato, Mexico; repreciado@yahoo.com; 3 Campo Experimental Valle del Fuerte, Instituto Nacional de Investigaciones Forestales, Agrícolas y Pecuarias, Guasave C.P. 81110, Sinaloa, Mexico; luispeinado@gmail.com

**Keywords:** specialty corn, phenolic acid, antioxidant, hypoglycemic

## Abstract

High-oil corn (HOC, >6% oil) is associated with increased germ size, which can be accompanied by greater accumulation of bioactive compounds such as phenolics. This study compared the phenolic content, antioxidant capacity (AC), and α-glucosidase inhibitory activity (αGI) of HOC and commercial corn hybrids. Twelve HOC hybrids (eight white, four yellow) obtained from doubled haploid lines and three normal corn hybrids (white) were used. Methanolic extracts were analyzed for total phenolics (Folin–Ciocalteu), flavonoids (AlCl_3_), AC (ABTS, DPPH), phenolic profiles by liquid chromatography–mass spectrometry, and αGI. All HOC hybrids met the test weight (≥73 kg/hL) required for nixtamalized products. They showed great variability in total phenolics, total flavonoids, and AC; the white HOC hybrid NWP19xNWP81 showed the highest values, significantly higher than those of commercial hybrids. Phenolic profiles showed 19 compounds, mostly ferulic acid derivatives; the NWP19xNWP81 hybrid showed the highest content of most of them. The levels of 15 compounds correlated positively with the AC. Eight HOC hybrids showed higher αGI than the commercial hybrids, highlighting BYP103xNYP135 (yellow). Some compounds (e.g., dehydrodiferuloyl diarabinofuranoside and dimethyl dehydrodiferuloyl diarabinofuranoside) showed high affinity for α-glucosidase. These results suggest that some HOC hybrids have superior nutraceutical quality, and their use as food or feed could provide better health benefits than normal corn.

## 1. Introduction

Corn (*Zea mays* L.) is the most important cereal crop worldwide with a global production of over 1.2 billion tons in 2024 [1]. It is used for human and animal nutrition, as well as for a variety of industrial applications. The development of high-oil corn (HOC) initiated with recurrent selection programs at the end of the 19th century, particularly the Illinois Long-Term Selection Experiment, which increased kernel oil content several-fold compared with conventional corn populations [2]. In Mexico, the National Institute of Forestry, Agriculture and Livestock Research (INIFAP) has white and yellow HOC doubled haploid (DH) lines derived from the Northwest White Population (NWP), the Bajío White Population (BWP), the Northwest Yellow Population (NYP), and the Bajío Yellow Population (BYP). These populations were improved for oil content (6.7–8.1%) following a recurrent half-sib selection scheme from 2004 to 2013 [3,4]. HOC DH lines have been used by INIFAP to produce experimental HOC hybrids, and some of them have already been released [5].

The inclusion of Mexican HOC DH hybrids represents a good alternative for improving grain nutritional quality. Ortíz-Islas, et al. [6] demonstrated the feasibility of developing HOC DH hybrids adapted to subtropical environments. The oil content of the hybrids ranged from 5% to 8%, and the values correlated positively with the germ size and protein content (10.2–16.5%). Consequently, there was an increase in the total content of the essential amino acids lysine and tryptophan, but not in terms of protein percentage. HOC hybrids also have a higher content of unsaturated fatty acids (oleic + linoleic) compared to normal corn [7]. Rodríguez-Treviño, et al. [8] reported that recurrent selection for oil content increased germ size and the contents of tocopherols and phytosterols. The positive association observed between oil content and liposoluble nutraceuticals may also hold for other bioactive components, such as phenolic compounds [9,10]. The germ contains the highest concentration of soluble phenolics, and the pericarp contains mainly insoluble phenolics [11]. The phenolic content in commercial corn showed a positive correlation with oil content [12]. In this regard, comparative gene expression analysis between ultra-high-oil and regular sweet corn suggested that fatty acid and phenylpropanoid biosynthesis are closely associated, and their accumulation may be influenced via carbon source reallocation [13].

Normal corn contains mostly phenolic acids and minor amounts of flavonoids [10,14]. Phenolic acids are mainly ferulic acid derivatives [15,16,17,18], which include hydroxycinnamic acid amides that have been involved in plant developmental processes, plant defense responses, and human health-related properties [19]. The analysis of 93 yellow corn varieties from China identified mainly *N′, N″*-diferuloyl putrescine and *N′, N″*-dicoumaryl spermidine in the free phenolic fraction and *trans*-*p*-coumaric acid, and *trans*- and *cis*-ferulic acid in the bound fraction [20]. A highly significant correlation was observed between the great variability in total phenolic content and the antioxidant capacity (ABTS, DPPH, and FRAP). A recent study with 233 maize inbred lines demonstrated a substantial genetic diversity and environmental stability in ferulic acid content. In addition, the levels of this compound were relatively stable to some common food-processing methods, highlighting the importance of maize genotypes as a source of bioactive compounds for the development of health-promoting products. Corn phenolics have also shown α-glucosidase inhibitory activity [21,22,23] and antidiabetic activity in rats [24]. However, there is little information about the accumulation of phenolic compounds and their potential biological activities in HOC. It is known that breeding for high-oil content improves the fatty acid profile and the protein content, which is associated with an increase in germ size [25,26,27]. Since phenolic content in normal corn shows a positive correlation with oil content [12] and antioxidant capacity [21], it could be expected that HOC genotypes contain a better phytochemical composition, representing promising materials from a nutritional and functional perspective. This study aimed to analyze the relationship between phenolic compound content, antioxidant capacity, and α-glucosidase inhibitory activity in HOC hybrids from Mexico.

## 2. Results and Discussion

### 2.1. Seed Physical Characteristics

The HOC and commercial corn hybrids showed significant differences in seed physical characteristics (Table 1). The hectoliter weight (HW) or bulk seed density varied from 76.8 to 81.5 kg/hL in the HOC hybrids. HW is a seed quality criterion established in the Official Mexican Standard NMX-FF-034/1-SCFI-2020 [28], and all HOC hybrids met the minimum density requirement (≥73 kg/hL) recommended for producing nixtamalized corn products [28]. In contrast, only one of the commercial hybrids (Armadillo) satisfied this recommendation (Table 1). These values agreed with those reported in four subtropical HOC populations (white and yellow) [4]. However, they were slightly higher than those registered for six HOC hybrids from Dupont US (73.2–75.8 kg/hL) [29].

The thousand-seed weight (1000-SW) varied from 260.8 to 398.2 g (Table 1) and fell within the range (228.3–447.9 g) reported by Ortíz-Islas, et al. [6] in 46 HOC DH hybrids grown in two locations of Central Mexico. The values were also close to those reported by Ignjatovic-Micic, et al. [30] for 13 HOC populations (273–350 g) from different countries, and by Pan, et al. [29] for six HOC hybrids (265.8–281.9) from Dupont, US.

The average kernel dimensions of the corn hybrids were 12.32 mm (length), 8.66 mm (width), and 4.36 (thickness). The seed dimensions of the HOC hybrids (Table 1) were similar to those registered previously by Ortíz-Islas, et al. [6] and Ignjatovic-Micic, et al. [30]. The 1000-SW parameter showed moderate positive correlations with seed length (r = 0.44, *p* < 0.01) and width (r = 0.46, *p* < 0.01) (Supplementary Table S1). Based on these parameters, the HOC hybrids with the smallest seeds are NWP81 × NWP19, NWP19 × NWP81, and NWP27 × NWP84.

### 2.2. Extraction Yield of Lipid and Phenolic Fractions

The HOC experimental hybrids showed significantly higher lipid yields (5.4–7.8%) than commercial corn hybrids (3.9–4.7%) (*p* ≤ 0.05), except for NWP13 × NWP85 and NWP32 × NWP9 (Table 2). The highest yield was observed in the NWP19 × NWP81 hybrid (7.85%). These values correspond with the range of oil content (5.5–8.7%) reported by Picón-Rico [31] in 56 diallel crosses of yellow HOC DH lines derived from the NYP and the BYP. Vázquez-Carrillo, et al. [32] also reported oil contents in white and yellow HOC genotypes (5.1–6.7%) similar to the lipid yields obtained in the present study (Table 2), except for the NWP19 × NWP81 hybrid (7.85%). However, the values of this study were considerably lower than those reported for ultra-high-oil corn grains (9.46–26.08%) [13]. The lipid yield showed a highly significant (r = 0.72, *p* < 0.001) positive correlation with HW (Supplementary Table S1), which is consistent with the higher seed density observed in HOC compared with normal corn (Table 1).

Many factors contribute to increased corn oil content, which is associated with larger germ size. The genetic analysis of HOC DH lines derived from subtropical populations of Mexico identified three molecular markers close to genes related to oil accumulation: FAX1, a protein that transports fatty acids from plastids, an LTP protein that enhances lipid transport between membranes, and a HXXXD-type acyltransferase [33]. Corn plants expressing wheat Puroindole (PIN) genes, which share a similar structure to non-specific LTPs, showed increased germ yield (33.8%) and seed oil content (25.23%) [26]. The Maize Giant Embryo (GE) gene (*ZmGE2*) belongs to the CYP78A subfamily, whose members have been associated with the regulation of organ size and the stimulation of cell proliferation, among other functions; the insertion of a transposon in *ZmGE2* increased the embryo-to-endosperm ratio and oil content [27]. Mutations in four corn genes (*bige1*–*bige4*) generate big embryos; *bige1* encodes a tonoplast inositol phosphate (InsP6) transporter, while *bige2*, *bige3*, and *bige4* encode inositol phosphate kinases. Several inositol phosphates participate as ligands of phytohormones (auxin and jasmonic acid) involved in cell proliferation, cell growth, and development; four of the five kinases are expressed in the embryo [25]. Thus, oil overproduction involves the adjustment of many metabolic pathways in corn.

The yields of the phenolic fractions from the HOC hybrids were very low (0.43–0.90%), but in general, they were higher than those of the commercial corn (0.52–0.64%). Six HOC crosses showed the highest values, including NWP19 × NWP81, which also showed the highest lipid yield (Table 2). These results suggest that the increase in oil content is also reflected in higher levels of phenolic compounds. Transcriptomic studies have shown that genes involved in phenolic biosynthesis are overexpressed during oil accumulation in corn. The gene expression of ultra-high-oil corn grains was compared with that of conventional sweet corn lines; as expected, the expression of fatty acid elongation pathway genes was upregulated, as well as those of secondary metabolism, including phenylpropanoid and flavonoid biosynthesis genes [13]. Corn kernel development is complex and involves many biochemical pathways, resulting in different endosperm and germ characteristics. During the middle–late phase of kernel development (~12–40 DAP), the endosperm is filled mainly with starch and proteins, and the germ with oil [34]. The expression of phenylpropanoid biosynthesis genes in corn developing kernels (15 to 48 DAP) revealed that phenylalanine ammonia lyase (*ZmPAL1*) and cinnamate 4-hydroxylase (*ZmC4H1*) were upregulated at an early stage of development (21 DAP) and coumarate CoA-ligase (*Zm4CL1*) at the late stage [10]. In the corn B73 inbred line, 19 genes were identified as the main regulators of phenolic metabolite flux, including *ZmPAL1*, *ZmC4H1*, and *Zm4CL1*. The highest expression of the core genes occurred mainly in the embryo from 0 to 14 DAP [9].

### 2.3. Total Phenolic and Flavonoid Content

The total phenolic (TP) content (mg EAG/100 g d.w.) ranged from 61.46 to 130.44 (Table 2). Six HOC crosses showed significantly (*p* < 0.05) higher TP values (98.19–130.44) than the commercial hybrids (74.72–81.20). NWP19 × NWP81 (white) showed the highest TP content, followed by NYP157 × NYP218 (yellow). As expected, TP content showed a positive correlation (r = 0.69, *p* < 0.001, Supplementary Table S1) with the yield of the phenolic fraction.

Das and Singh [35] reported that corn bran (pericarp and aleurone) contains the highest concentration of phenolics, followed by the germ; however, the germ occupies a higher proportion of the kernel weight than the bran and has a larger contribution to the phenolic content. These authors also found a positive correlation between germ size (%) and phenolic content. Thus, an increase in TP was expected in the HOC hybrids [9,10]. In fact, the lipid fraction and the TP content showed a positive correlation (r = 0.69, *p*< 0.001, Supplementary Table S1). In this regard, Mahan, et al. [12] also reported a positive correlation between oil content (1.4–5.6%) and TP (17.1–69.0 mg EAG/100 g d.w.) in 84 corn hybrids obtained from diallelic crosses of 11 parents (red, blue, purple, and yellow). This association may result from the simultaneous synthesis of phenolics and oil during embryo development, as suggested by comparative gene expression analysis between ultra-high-oil and regular sweet corn, which indicates the upregulation of fatty acid biosynthesis and phenylpropanoid biosynthesis genes [13]. Both pathways may coordinate to support maturation, storage, and protection against oxidative stress. These results indicate the potential of HOC as a source of bioactive phenolic compounds.

The total flavonoid (TF) content (mg EC/100 g d.w.) of the HOC hybrids (24.16–29.97) was similar to that of the commercial hybrids Garañon (30.8) and P3140W (28.23) (Table 2). These values are similar to those observed in white (24.9 mg EC/100 g d.w.) and yellow (28.1 mg EC/100 g d.w.) corn inbred lines, probably attributable to flavonols and flavones that contribute to the cream and yellow color of the kernels; nevertheless, flavonoid-type compounds were not identified by HPLC in these lines [14]. On the other hand, Djalovic, et al. [36] reported considerably higher TF values, as equivalents of quercetin (EQ), for hybrid corn (68.7–157.8 mg EQ/100 g d.w.); however, it has been suggested that using quercetin as a reference standard in the NaNO_2_-AlCl_3_ assay overestimates the results [37]. In contrast, lower TF contents were obtained in the soluble fraction (SF) and insoluble fraction (IF) of white corn (1.47, 11.9 mg EC/100 g d.w.) and yellow corn (2.1, 14.5 mg EC/100 g d.w.) using this method [38]. Zhang, et al. [10] reported that TF levels (mg EC/100 g d.w.) of yellow corn decrease gradually during kernel development.

The differences in the content and type of metabolites in the HOC hybrids may be related to the extraction method used. The analysis of TP content (mg FAE/100 g d.w.) in tortillas from an INIFAP HOC (Bajío, México) showed a lower value in the free fraction (33.3) with respect to the bound fraction (143.1) [39]. The TF content (mg EC/100 g d.w.) of white and yellow corn genotypes from Mexico was lower in the SF (8.25 and 10.5) than in the IF (15.75 and 48.75) [40]. Direct acid hydrolysis of defatted flour would yield a mixture of FS and FI; however, acid hydrolysis has been reported to be less effective at breaking the ether and ester bonds linking phenolic compounds to cell wall components [41]. In addition, acidic conditions can degrade some phenolic compounds such as flavonols and ferulic acid, depending on the plant matrix [41]. Acid hydrolysis is recommended for the recovery of hydroxycinnamic acids bound to arabinoxylans [42].

The NWP19 × NWP81 cross showed higher values of lipid fraction, phenolic fraction, TP, and TF than those in the NWP81 × NWP19 reciprocal cross (Table 2), suggesting that these traits are favored when NWP19 is used as the female parent. However, data from parental lines are required to establish if there is a maternal effect. Furthermore, this effect was not registered for the reciprocal crosses between the NYP135 and BYP103 lines.

### 2.4. Phenolic Profiles of High-Oil Corn Extracts

**Figure 1:**
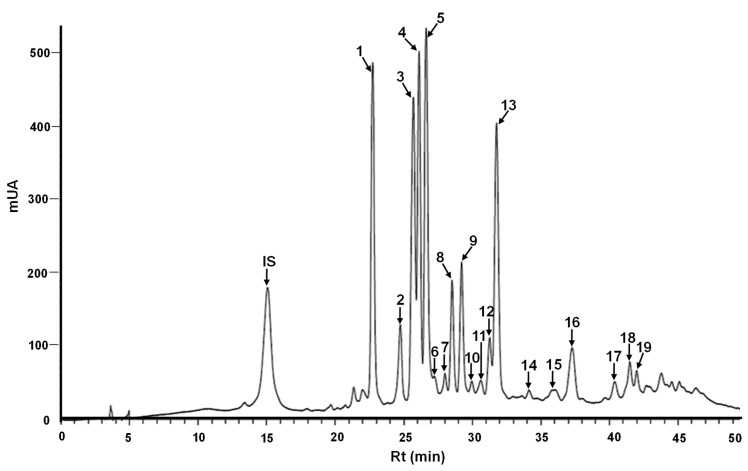
Representative UPLC-DAD chromatographic separation of methanol extracts from mature seeds of high-oil corn hybrids. Peak identities are shown in Table 3. Gallic acid was used as the internal standard (IS).

The 15 corn hybrids analyzed by UPLC-DAD showed a similar phenolic compound profile (Figure 1), characterized by 19 peaks with UV spectra corresponding to phenolic acids, whereas flavonoids were not detected. These results correspond with those of Žilić, et al. [14], who did not detect flavonoids by HPLC in white and yellow corn inbred lines despite the colorimetric quantification of total flavonoids.

The peak identities were determined by mass spectrometry (MS) (Table 3) based on the molecular ion [M−H]^−^ fragmentation pattern data reported in the literature, and MS data from ferulic acid, *p*-coumaric acid, and tryptophan standards. The identified phenolics belong to hydroxycinnamic acid derivatives, such as ferulic and *p*-coumaric acids. Ferulic acid is the main phenolic compound in corn and is predominantly found in bound forms within cellulose and lignin [43]. The arabinoxylan conjugated hydroxycinnamic acids included six isomers of dehydroferuloyl diarabinofuranoside (DFA-MeAra2), five isomers of *bis-N,N’*-diferuloyl putrescine (Bis-DFP), other compounds such as *p*-coumaroyl-feruloyl putrescine (*p*-CFP) and *N,N’*-coumaroyl feruloyl putrescine (NN-CFP), and methyl 5-*O*-feruloyl arabinofuranoside (FA-MeAra), as well as the presence of the dipeptide tyrosyl-tryptophan.

Amino-substituted hydroxycinnamic acid derivatives (e.g., ferulic acid and *p*-coumaric acid) are widely distributed in plants. They are synthesized via the phenylpropanoid pathway and found in different plant organs. In corn, these compounds are conjugated with polyamines, involved in several processes in the plant, including stress responses, antimicrobial activity, and potential health benefits [19]. Peptides have been associated with diverse biological activities (e.g., antimicrobial, immunomodulatory, antihypertensive, antioxidant), and those with antioxidant activity are important because chronic degenerative diseases are associated with oxidative stress, and antioxidant peptides could be helpful in their prevention and treatment. Peptides containing Tyr and Trp exhibit antioxidant activity and are strong radical scavengers [44]. Thus, tyrosyl-tryptophan could be an important contributor to the biological activities of the corn extract.

The compounds identified in the HOC hybrids (Table 3) coincide with those characterized (LC-MS/MS) by several authors in normal corn, such as those reported by Bento-Silva, et al. [15] in whole grain corn flour and Lux, et al. [18] in different experimental hybrids of German yellow corn, who reported the identification of *bis-N,N’*-diferuloylputrescine (bis-DFP) and *N,N’*-coumaroylferuloylputrescine (NN-CFP), among others. LeClere, et al. [17] reported that DFP and CFP accumulate predominantly in the pedicel and to a lesser extent in the pericarp during corn kernel development.

**Figure 2:**
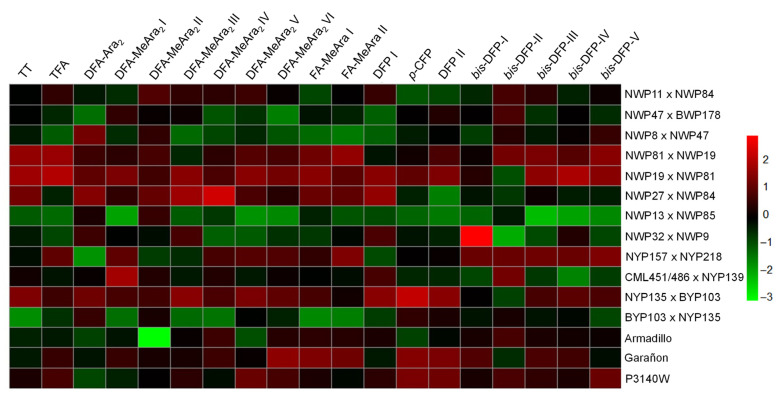
Heatmap of metabolites identified by UPLC-DAD-MS in high-oil corn. Each column represents a metabolite, and each row represents a maize hybrid. The heatmap scale bar shows standardized metabolite content values color-coded from low (green) to high (red). TT, tyrosil-tryptophan; TFA, dehydrotriferulic acid, hydrated; DFA-MeAra2, dimethyl dehydrodiferuloyl diarabinofuranoside I, II, III, IV, V, VI; Fa-MeAra, methyl 5-*O*-feruloyl arabinofuranoside I, II; DFA Ara2, dehydrodiferuloyl diarabinofuranoside; *p*-CFP, *p*-coumaroyl-feruloyl putrescine; DFP, *N,N’*-diferuloyl putrescine; Bis-DFP, *bis-N,N’*-diferuloyl putrescine I, II, III, IV, V.

A heatmap was used to visualize the variation in metabolite content among the corn hybrids (Figure 2). The white HOC hybrid NWP19 × NWP81 showed the highest levels of most of the compounds identified, which corresponds with the fact that this cross also showed the highest yields in oil and phenolic fractions, as well as the highest content of total phenolics. The white reciprocal cross NWP81 × NWP19 also showed high levels of several compounds, followed by NWP27 × NWP84, whereas NWP13 × NWP85 exhibited the lowest abundance of most of the compounds. Regarding the yellow HOC hybrids, NYP157 × NYP218 and NYP135 × BYP103 showed high concentrations of several of the quantified metabolites. DFA-MeAra2 I, FA-MeAra I, FA-MeAra II, and Bis-DFP II were the most abundant compounds (Supplementary Table S2).

### 2.5. Antioxidant Capacity

The antioxidant capacity (µmol TE/100 g d.w.) measured by the DPPH method was higher in the two HOC hybrids (383.04–780.39) than in the commercial hybrids (511.01–608.44), with the highest values observed in the white cross NWP19 × NWP81 (DPPH = 780.39; ABTS = 2498.58) and the yellow cross NYP157 × NYP218 (DPPH = 716.06; ABTS = 2218.40) (Table 4). On the other hand, the antioxidant capacity values (µmol ET/100 g d.w.) obtained by the ABTS method were on average 3.3 times higher than those obtained by the DPPH method. However, most of the HOC hybrids showed ABTS values similar to those of commercial corn. As observed for DPPH, the highest ABTS values were registered for the white cross NWP19 × NWP81 (2498.58) and the yellow cross NYP157 × NYP218 (2218.40) (Table 4). For both methods, the average values of the yellow HOC crosses (DPPH = 681, ABTS = 2242) were higher than those of the white crosses (DPPH = 543, ABTS = 1752), although the highest values were observed in the white cross NWP19 × NWP81 (Table 4). The TP and antioxidant capacity were positively correlated (DPPH, r = 0.77; ABTS, r = 0.78; *p* ≤ 0.001), while the levels of 15 out of the 19 identified phenolics were also positively correlated with the antioxidant capacity measured by both methods (*p* ≤ 0.01) (Supplementary Table S1).

The antioxidant capacity of soluble and insoluble maize phenolic extracts has been extensively studied. Zhang, et al. [10] showed that during corn kernel development, the TP and antioxidant activity correlated positively (*p* < 0.05, r = 0.71), observing an increase in both parameters from 15 DAP (TP = 242 mg EAG/100 g d.w.; ABTS = 19 μmol TE/g d.w.) to 48 DAP (TP = 304 mg EAG/100 g d.w.; ABTS = 35 μmol TE/g d.w.). González-Muñoz, et al. [21] studied 33 accessions of native Chilean corn and showed that the total antioxidant capacity (µmol ET/100 g d.w.) of white and yellow corn accessions was 839 and 757 (DPPH), and 1508 and 1587 (ABTS), respectively; the values of both methods correlated with each other and with the TP content (mg EAG/100 g d.w.) (white 176.7, yellow 187.8). In the present study, the NWP19 × NWP81 HOC hybrid showed higher antioxidant values than those reported by González-Muñoz, et al. [21]. In addition, the antioxidant capacity correlated positively with most of the identified phenolics (Supplementary Table S1). It should be noted that although the TP content reported for the Chilean corn genotypes nearly doubles that observed in the present study, the antioxidant capacity measured by ABTS was higher in the HOC hybrids, suggesting that these materials contain individual phenolics with greater antioxidant capacity or specific mixtures acting synergistically. The reported compounds in the Chilean corn were hydroxycinnamic acids (i.e., ferulic, coumaric, caffeic), protocatechuic, vanillic, and vanillin, compounds recognized for their antioxidant capacity [21]. The compounds identified in the present study were mostly conjugated hydroxycinnamic acids (e.g., arabinofuranoside, putrescine) (Table 3), which could increase their antioxidant activity and improve their stability under physiological conditions and during oral administration [19]. Zhang, et al. [20] also identified conjugated hydroxycinnamic acids in 93 yellow corn varieties from China, and the total phenolic content showed a highly significant correlation with the antioxidant capacity (ABTS, DPPH, and FRAP).

### 2.6. Inhibitory Activity of the α-Glucosidase Enzyme

**Figure 3:**
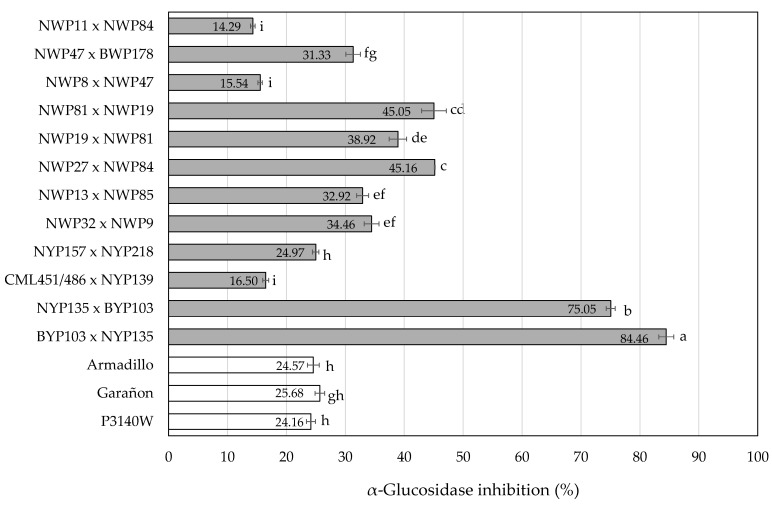
α-glucosidase inhibition (αGI) of the phenolic fraction of high-oil corn (HOC) hybrids. Mean ± standard deviation (three replicates). The samples were evaluated at a concentration equivalent to 166.67 mg flour d.w./mL. Means with different letters are significantly different (Tukey, *p* ≤ 0.05).

The α-glucosidase (αGI) inhibition at the concentration evaluated (166.67 mg flour d.w./mL) ranged from 14.29 to 84.46% (Figure 3). Compared with the αGI of the commercial hybrids, seven out of the 12 HOC hybrids showed significantly higher activity, highlighting the yellow reciprocal crosses BYP103 × NYP135 (84.46%) and NYP135 × BYP103 (75.05%). α-glucosidase inhibitors such as acarbose are used to control postprandial hyperglycemia in diabetic patients. This compound was used as a positive control (1 mg/mL), and the αGI was 45.45%. Considering the extraction yields of the yellow HOC hybrids NYP135 × BYP103 (0.69% d.w.) and BYP103 × NYP135 (0.72% d.w.), the concentrations of the phenolic fractions evaluated were 1.15 and 1.20 mg/mL, respectively, which were comparatively more active than acarbose. In contrast, the αGI activity of ethanolic extracts (2 mg extract/mL) from white (≈30%), yellow (48%), and pigmented (20–55%) native Mexican corn was lower than that of acarbose (62%, 1.76 mg/mL) [22].

In the search for relevant hypoglycemic agents, it is desirable that they show equal or greater αGI than acarbose and low inhibitory activity against α-amylase. This selectivity is crucial, since the adverse gastrointestinal effects of acarbose are attributed to its strong inhibitory activity against α-amylase [45]. In this regard, several authors report that the bound phenolic fractions do not exhibit inhibitory activity against these enzymes. In contrast, free phenolics exhibit high αGI and show negligible-to-moderate activity against α-amylase [21,46].

For the 33 accessions of native Chilean corn [21], all free phenolic extracts (125 mg d.w./mL) inhibited yeast α-glucosidase, and the inhibition percentages (19–72%) were similar to those obtained in the HOC hybrids (166.66 mg d.w./mL) (24.97–84.46%) (Figure 3). Gálvez Ranilla, et al. [46] also showed similar inhibition percentages (32.5–76.1%) of yeast α-glucosidase for free phenolic extracts (125 mg d.w./mL) from 22 Peruvian corn samples corresponding to five corn landraces (Arequipeño, Cabanita, Kculli, Granada, and Coruca). In both studies, HPLC analysis of the bound phenolic fraction (alkaline hydrolysis) showed mainly ferulic, coumaric, and caffeic acids. In contrast, the free phenolic fraction showed a greater number of peaks corresponding to conjugated forms of phenolic acids, which were associated with αGI activity [21]. This is consistent with the findings of Niwa, et al. [47], who performed bio-targeted fractionation of the ethanolic extract of corn gluten and identified phenolic acid amides as the compounds responsible for αGI. In their evaluation, the compound *N,N’*-coumaroyl feruloyl putrescine (NN-CFP) showed twice the activity of *N,N’*-diferuloyl putrescine. Both compounds were also identified in the HOC hybrids from this study.

Other studies have not reported a correlation between αGI activity and TP content or antioxidant capacity [21,47,48]. However, this study showed a slight correlation between the αGI activity and the antioxidant capacity by ABTS (r = 0.31, *p* ≤ 0.05) (Supplementary Table S1), and a slight-to-moderate positive correlation with the levels of five phenolics: DFA-Ara_2_, DFA-MeAra_2_ III, DFP I, *p*-CFP, and DFP II (*p* ≤ 0.05) (Supplementary Table S1). Thus, these five compounds could contribute more to αGI activity. Specifically, compounds such as NN-CFP and DFP have shown inhibitory effects against αGI [47], suggesting their potential use for the treatment of hyperglycemia.

The chemical extraction methods for phenolic compounds (acid or alkaline hydrolysis) are aggressive, and it has been suggested that they lack physiological relevance because the biological activities rely on release–absorption processes [41]. In this study, the acid hydrolysis conditions used, the types of compounds extracted, and the concentration obtained suggest that these compounds could be released from the food matrix by gastric fluids and act as α-glucosidase inhibitors in the small intestine.

Many studies have demonstrated the biological relevance of the α-glucosidase inhibitory activity because the compounds act in the gut lumen. Thus, the in vitro assay provides valuable data for the in vivo activity. For example, an extract of *Xylocarpus mekongensis* enriched in six major phenolics (catechin, catechol, (-)-epicatechin, syringic acid, *trans*-ferulic acid, and *trans*-cinnamic acid) was a potent inhibitor of α-glucosidase (IC_50_ = 0.420 mg/mL), and in diabetic mice, the extract (500 mg/kg b.w.) reduced the hyperglycemic peak, showed antidiabetic activity, and normalized the hepatic and renal markers, with similar results to those obtained with glibenclamide [49]. The main compounds in the essential oil of *Teucrium poium* are phenolics (carvacrol and thymol); the oil inhibited α-glucosidase (73% at 0.2 mg/mL) similarly to acarbose, and its oral administration (70 mg/kg b.w.) to diabetic rats restored the glucose and biochemical parameters [50]. In this study, the extracts of some HOC materials had better α-GI activity than acarbose, and these genotypes may contribute to diabetes management.

Molecular docking analysis was performed to predict the interactions and affinity for α-glucosidase of the phenolic compounds whose content correlated with the αGI activity. The binding energies of most of the phenolics evaluated were lower than that of acarbose (Table 5), suggesting a good affinity of these compounds for α-glucosidase. The highest affinity for the enzyme was observed for DFA-Ara_2_. This compound established multiple hydrogen bonds with key catalytic residues of α-glucosidase, including Asp518 and His674 (Supplementary Figure S1).

The phenolic extract of the experimental HOC hybrid with the second-highest αGI value (NYP135 × BYP103) (Figure 3) showed high levels of the compounds evaluated in the docking analysis (Figure 2). However, these phenolics were considerably less abundant in the HOC hybrid with the highest αGI value (BYP103 × NYP135), suggesting a complex interaction among the compounds present in the extract. In addition, the predicted affinity does not necessarily correspond with the in vitro or in vivo activity. For instance, Niwa, et al. [47] reported that the αGI activity of *p*-CFP was almost three times higher than that of DFP, while DFP showed a slightly better affinity than *p*-CFP (Table 5). Nevertheless, these discrepancies can be attributed to differences between the enzymes used in the in vitro assay and those used in the in silico analysis or to the fact that the docking analysis was directed to the active site and the compounds may be inhibiting the enzyme by mixed and non-competitive mechanisms [51].

### 2.7. Association Between the Characteristics Evaluated in the High-Oil Corn Hybrids

Network correlation analysis (*r* ≥ 0.4) showed the associations among phenolics, antioxidant capacity, and α-glucosidase inhibition (Supplementary Figure S2). This analysis revealed three clusters indicated by different colors. Total flavonoid content was set apart and showed only a weak correlation with DFA-MeAra2 II, while α-glucosidase inhibition showed only two connections with the phenolic compounds DFA-Ara2 and *p*-CFP; the first compound showed the highest affinity for α-glucosidase in the docking analysis (Table 5). The other two clusters showed stronger correlations among the variables, highlighting the associations between the antioxidant capacity by DPPH and ABTS with the content of hydroxycinnamic acid amides (*p*-CFP, DFP, and Bis-DFP) and feruloyl arabinofuranosides (FA-MeAra and DFA-MeAra2).

**Figure 4:**
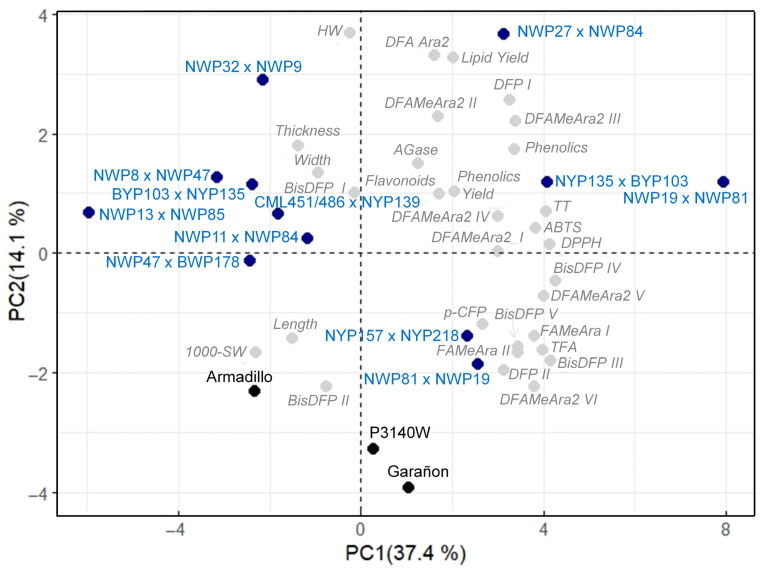
Principal component analysis based on physical characteristics, lipid content, phenolic content, antioxidant capacity and α-glucosidase inhibition of high-oil corn hybrids. The color codes used are as follows: HOC hybrids in blue, commercial hybrids in black, and variables in gray. TT: Tyrosil-tryptophan; TFA: Dehydrotriferulic acid, hydrated; DFA-MeAra_2_: Dimethyl dehydrodiferuloyl diarabinofuranoside I, II, III, IV, V, VI; Fa-MeAra: Methyl 5-O-feruloyl arabinofuranoside I, II; DFA-Ara_2_: Dehydrodiferuloyl diarabinofuranoside; *p*-CFP: *p*-Coumaroyl-feruloyl putrescine; DFP: *N,N’*-Diferuloyl putrescine I, II; *Bis*-DFP: *bis*-*N,N’*-Diferuloyl putrescine I, II, III, IV, V; HW: hectoliter weight; Agase: α-glucosidase inhibition; ABTS and DPPH: antioxidant capacity methods.

Principal component analysis (PCA) showed that two components explained 51.5% of the total variation in the HOC and commercial hybrids (Figure 4). The main contributors to component 1 (PC1, 37.4%) were the antioxidant capacities evaluated by DPPH and ABTS, TP, and the content of several metabolites (TT, TFA, FA-MeAra, DFA-MeAra2, *p*-CFP, DFP, Bis-DFP III-V). The variation explained by the principal component 2 (PC2, 14.1%) was mainly associated with HW, lipid fraction, and DFA-Ara2 content. Consistent with the contribution of PC1, most of the compounds were clustered in the upper-right and lower-right quadrants of the biplot, but they did not show clear associations with the hybrids. The HOC hybrids were divided into three quadrants, three in the upper-right quadrant that were superior for most of the characteristics, two in the lower-right quadrant with good content of some phenolics (e.g., TFA, Fa-MeAra, and *bis*-DFP), and seven in the upper-left quadrant that showed moderate-to-low values of several of the characteristics evaluated. The commercial corn hybrids were placed in the lower-left and lower-right quadrants, consistent with their lower performance in most of the characteristics. Thus, the PCA biplot clearly separated the experimental HOC hybrids from the commercial hybrids, and the clustering patterns provide relevant information for genotype selection. Based on this analysis, the most outstanding genotypes were the white HOC hybrids NWP19 × NWP81 and NWP27 × NWP84, and the yellow HOC hybrid NYP135 × BPY103 (Figure 4).

Some limitations of the current study include the use of plant materials grown in a single season at one location, as well as a phenolic extraction procedure that may underestimate the contributions of other fractions. Nevertheless, the substantial variability in phenolic content and composition, antioxidant capacity, and α-glucosidase inhibition among the experimental HOC hybrids indicates broad genetic diversity in these materials. This corresponds with the wide genetic variability reported for the parental HOC DH lines [52] and highlights their potential for breeding high-oil corn with enhanced nutritional and functional properties. HOC hybrids have been shown to be suitable for the elaboration of nixtamalized flours and tortillas [7]. All HOC hybrids used in the present study showed test weights > 73 kg/hL and may be suitable to produce nixtamalized products with higher nutritional and nutraceutical value than those produced from normal corn. However, additional studies are required to evaluate the effects of technological processes, such as nixtamalization, on HOC phenolics and their biological properties.

## 3. Materials and Methods

### 3.1. Reagents and Solvents

All reagents, including butylated hydroxytoluene (BHT), hydrochloric acid, Folin–Ciocalteu reagent, aluminum chloride, sodium nitrite, gallic acid, catechin, ferulic acid, coumaric acid, tryptophan, 1,1-diphenyl-2-picrylhydrazyl (DPPH), 2,2′-azino-bis(3-ethylbenzothiazoline)-6-sulfonic acid (ABTS), α-glucosidase from *Saccharomyces cerevisiae*, *p*-nitrophenyl glucopyranoside, 6-hydroxy-2,5,7,8-tetramethylchroman-2-carboxylic acid (Trolox), and acarbose were of analytical grade and purchased from Sigma-Aldrich (St. Louis, MO, USA). HPLC-grade and MS-grade organic solvents were obtained from Baker Inc. (Phillipsburg, NJ, USA) and Thermo Fisher Scientific Inc. (Waltham, MA, USA), respectively.

### 3.2. Plant Materials

Twelve experimental hybrids of high-oil corn (HOC) from the National Institute of Forestry, Agriculture and Livestock Research (INIFAP) and the commercial hybrids P3140W (Pioneer), Armadillo (Asgrow), and Garañon (Asgrow) were used. The experimental hybrids were generated by crossing doubled haploid (DH) lines obtained from the Northwest White Population (NWP), the Northwest Yellow Population (NYP), the Bajío White Population (BWP), and the Bajío Yellow Population (BYP) [3]. A triple cross was obtained using as the female parent a single cross of normal maize lines (CML451/486) from the International Maize and Wheat Improvement Center (CIMMYT), and as the male parent NYP139, a HOC DH line. All the materials were grown during the autumn–winter season of 2019–2020 at the experimental field of INIFAP in Juan José Ríos, Sinaloa, México (25°45′54.3″ N, 108°48′39.9″ W), according to the technical guide established by this Institute [53]. A randomized complete block design with three replications was used. Each plot consisted of four 10 m long rows placed 0.8 m apart. The average minimum and maximum temperatures were 12.9 °C and 29.4 °C. Mature seeds were harvested and stored in sealed plastic containers at −20 °C until use. For extraction of the phenolic fraction, the seeds were processed with a ball mill (Retsch MM400, Haan, Germany) to obtain flours that passed a 60-mesh sieve. Flour moisture was determined as indicated in the official method 925.09B [54].

### 3.3. Seed Physical Characteristics

The thousand-seed weight (g) and hectoliter weight (HW, kg/hL) were determined according to the Official Mexican Standard NMX-FF-034/1-SCFI-2020 [28] with an analytical balance (CP2245, Sartorius, Göttingen, Germany). The dimensions (mm) of 100 seeds (length, width, and thickness) were measured using a digital micrometer 3109-25A (INSIZE, Suzhou China) with 0.001 mm resolution. All measurements were made by triplicate.

### 3.4. Extraction of the Phenolic Fraction

The phenolic fraction was obtained using acid hydrolysis as reported by Das and Singh [35] and Bento-Silva, et al. [42]. The flour sample (500 mg) was first defatted using hexane (1:20 *w*/*v*, containing 4 µg/mL BHT) with ultrasonic agitation for 60 min at 30 °C. The mixture was centrifuged at 10,000 rpm for 5 min, the supernatant was recovered, and the residue was extracted one more time under the same conditions. The hexane phases were combined, and the solvent was removed to obtain the lipid fraction. The defatted flour was placed at 50 °C for 20 min to remove any remaining hexane, then hydrolyzed with 10 mL of methanol:2 M HCl (1:1 *v*/*v*) at 85 °C for 2 h, vortexing (10 s) the sample every 30 min. After the sample was cooled, the pH was adjusted to 2 with 2 M NaOH; then, 20 mL of water was added, and the mixture was centrifuged at 10,000 rpm for 5 min. The supernatant was carefully extracted with EtOAc (4 × 10 mL). The extracts were combined, dried with anhydrous Na_2_SO_4_, and the solvent was removed to obtain the phenolic fraction. The residues were dissolved in 1 mL of methanol (500 mg of flour per mL), filtered using a PVDF membrane (0.45 µm), and stored at −20 °C in the dark. The extractions were performed in triplicate.

### 3.5. Determination of Total Phenolics

Total phenolic content was determined using the Folin–Ciocalteu assay [55] adapted to microplates. In each well, 10 µL of phenolic fraction or standard was mixed with 100 µL of Folin–Ciocalteu reagent diluted in water (1:10). The microplate was shaken, left to stand for 2 min at room temperature, and then 90 µL of 10% Na_2_CO_3_ solution was added. The samples were kept at 40 °C for 30 min, and finally, the absorbance was measured at 765 nm (Synergy HTX, Biotek, Winooski, VT, USA). As color correctors, the samples were treated the same way, but with water rather than Folin–Ciocalteu reagent. A gallic acid curve (0–400 µg/mL) was used for quantification, and the results were reported as milligrams of gallic acid equivalents (mg GAE) per 100 g of dry weight (mg GAE/100 g d.w.).

### 3.6. Determination of Total Flavonoids

Total flavonoids (TF) were quantified spectrophotometrically as complexes with aluminum chloride. The methodologies of Jia, et al. [56] and Dewanto, et al. [57] were adapted for use in a microplate. In total, 20 µL of the sample (phenolic fraction or standard), 50 µL of water, and 100 µL of 0.5% NaNO_2_ were mixed. The microplate was shaken, and after 6 min at room temperature, 24 µL of 5% AlCl_3_ was added. It was kept at room temperature for another 5 min, and then 40 µL of 1 M NaOH was added. The microplate was vigorously shaken and left to stand for 15 min before the absorbance was measured at 510 nm (Synergy HTX, BioTek, Winooski, VT, USA). Water was used instead of AlCl_3_ as a color corrector for the samples. Quantification was performed using a catechin curve (0–500 µg/mL), and the results were reported as milligrams of catechin (mg CE)/100 g d.w.).

### 3.7. Phenolic Profiles

For the separation of the compounds, 500 μL of phenolic fraction was first mixed with 15 μL of internal standard (gallic acid, 1 mg/mL); the mixture was sonicated for 5 min, and then 5 μL was injected into an ACCELA UPLC-DAD (Thermo Fisher Scientific, Inc, Waltham, MA, USA). The separation was performed on a Luna C18 column (5 μm, 150 × 3 mm) (Phenomenex, Torrance, CA, USA) using water: 1% formic acid (Solvent A) and acetonitrile (Solvent B) as mobile phases at a flow rate of 0.2 mL/min. An elution gradient was used, starting with 99.5% Solvent A and 0.5% Solvent B, reaching 40% A and 60% B at minute 45. Finally, the column was equilibrated with 100% Solvent A for 5 min with a total sequence time of 50 min. Detection was performed at 280, 320, and 350 nm. After UPLC-DAD separation, the compounds in the phenolic fraction were analyzed using an LTQ-XL mass spectrometer (Thermo Fisher Scientific Inc.) with an electrospray ionization source operating in positive/negative mode at 35 V and 300 °C, respectively. Data acquisition was performed using Xcalibur software version 2.2 (Thermo Fisher Scientific Inc.) in full-scan mode, covering an *m*/*z* range of 110 to 2000. Helium was used for collision-induced dissociation (10–45 V) in MS^n^ experiments and nitrogen for drying. These analyses provided the molecular weight and fragmentation pattern of each compound.

The compounds were identified by comparing their UV spectra and MS fragmentation patterns with those reported in the literature and MS data obtained from commercial standards (i.e., ferulic acid, *p*-coumaric acid, and tryptophan). The quantification was carried out with calibration curves of the same standards.

### 3.8. Evaluation of Antioxidant Capacity

The antioxidant capacity was evaluated using the DPPH (Brand-Williams, et al. [58]) and ABTS [59] methods adapted to microplates. For DPPH, 20 µL of the sample (methanol [reaction control, Crxn], phenolic fraction [diluted 1:10 in methanol], or Trolox [12.5–200 µg/mL]) and 180 µL of DPPH radical in methanol (150 µM) were mixed; methanol was added instead of the radical as a blank (color corrector for each sample). The microplate was then incubated for 30 min at 37 °C, and absorbance was read at 515 nm (Synergy HTX, Biotek, Winooski, VT, USA).

For the ABTS method, the radical was diluted with PBS (10 mM, pH 7.4) until an absorbance of 1.4 ± 0.1 at 743 nm was obtained (BioMate 3S, Thermo Scientific, Madison, WI, USA). In each well, 20 µL of the sample (PBS [reaction control, Crxn], phenolic fraction [diluted 1:40 in PBS], or Trolox [6.25–100 µg/mL]) and 180 µL of ABTS radical were added. PBS was added instead of radical as a blank (color corrector for each sample). The microplate was incubated (37 °C/10 min), and the absorbance was read at 734 nm (Synergy HTX, Biotek, Winooski, VT, USA). For both methods, the percentage inhibition (%INH) was calculated from the absorbances using the formula

% INH = [((Abs Crxn − Abs Brxn) − (Abs m-Abs Bm))/(Abs Crxn − Abs Brxn)] × 100;

where Crxn = reaction control; Brxn = reaction blank; m = sample; Bm = sample blank.

Trolox equivalents (TEs) were calculated using the Trolox standard curve (% INH vs. Trolox concentration) and the results were reported as µmol TE/100 g d.w. The evaluations were performed for each of the three phenolic fractions.

### 3.9. Inhibition of α-Glucosidase

It was evaluated using yeast α-glucosidase and *p*-nitrophenylglucopyranoside as substrate. For the assay, 120 µL of each phenolic fraction was dried and resuspended in an equal volume of phosphate buffer (PBS 0.1 M, pH 6.9). The evaluation was performed using a microplate according to López-Angulo, et al. [60]. First, 25 µL of the sample (phenolic fraction [500 mg d.w./mL], acarbose [3 mg/mL], or PBS [reaction control, Crxn]) was mixed with 50 µL of α-glucosidase (0.5 U/mL) in PBS, and incubated for 10 min at 37 °C. The equivalent concentrations of flour and acarbose in the presence of the enzyme were 166.67 and 1 mg/mL, respectively. Then, 25 µL of the substrate *p*-nitrophenylglucopyranoside (5 mM in PBS) was added, except in the color corrector wells (sample blank), where PBS was added. The microplate was incubated (37 °C/10 min), and the absorbance was measured at 405 nm (Synergy HTX, Biotek, Winooski, VT, USA). The results were expressed as percentages of inhibition, calculated as described in the antioxidant capacity Section 3.8.

### 3.10. Molecular Docking Analysis

The 3D structures of the phenolic compounds were generated using Marvin JS (https://marvinjs-demo.chemaxon.com/latest/demo.html accessed on 30 March 2026). They were prepared in MOE v.2014.09.01 (Chemical Computing Group, Montreal, Canada) by adding hydrogen atoms, assigning Gasteiger partial charges, and automatically correcting valence states, molecular geometry, and the orientation of hydrogen atoms and lone pairs. Energy minimization was performed using the MMFF94 force field under non-periodic conditions with full ligand flexibility until a root-mean-square (RMS) gradient convergence criterion of 0.1 kcal/mol/Å^2^ was reached, generating energetically stable conformations for docking analysis. The structure of the α-glucosidase (PDB ID: 5NN8) protein was obtained from the Protein Data Bank (https://www.rcsb.org/ accessed on 30 March 2026), and non-standard molecules and water molecules were removed. Protein preparation and molecular docking were performed using the Dock protocol available in MOE. Protein preparation was conducted using the Structure Preparation protocol (Compute, Structure Preparation, Protonate3D, and Correct), where structural inconsistencies were corrected, missing hydrogen atoms were added, and the protein was protonated under physiological conditions at 310 K. The analysis was directed to the active site of the enzyme, which is formed mainly by the acid residues Asp518, Glu521, and Asp616, as well as the basic residues Arg600 and His674. Ligand placement was carried out using the Triangle Matcher algorithm, followed by initial rescoring with the London dG scoring function, retaining the top 100 poses for refinement using the force field method and rescoring with the GBVI/WSA dG scoring function. The top 30 poses were retained, and duplicate conformations were removed during rescoring. Final docking poses were selected according to the most favorable S score (binding affinity energy, kcal/mol) and interactions with catalytic residues in the active site.

### 3.11. Statistical Analysis

Data from three replicates were analyzed by one-way ANOVA. The Tukey test (α = 0.05) was used to compare the means among the genotypes. Pearson’s correlation analysis was also carried out using the software STATGRAPHICS Plus version 5.1 (Statistical Graphics Corporation™, Warrenton, VA, USA). Principal component analysis (PCA) was conducted to examine the relationships among variables evaluated in the corn hybrids using RStudio version 2024.12.1 (R Foundation for Statistical Computing, Vienna, Austria). FactoMineR and factoextra packages were used for PCA and visualization, respectively. Normalization of individual compound levels via logarithmic transformation (log2) and autoscaling were applied for heatmap analysis using the heatmap function of gplots package in R. Cluster analysis of network correlation was conducted from variables showing a positive correlation with r ≥ 0.4 using the ggraph and tidygraph packages in R.

## 4. Conclusions

The HOC hybrids showed great variability in phenolic content and composition, antioxidant capacity, and α-glucosidase inhibition, with some of them showing higher values than those of commercial corn hybrids. Of the 19 compounds identified in the phenolic fraction, 18 were ferulic acid derivatives, and 17 of them correlated with the antioxidant capacity. Some compounds showed high affinity for α-glucosidase (e.g., dehydrodiferuloyl diarabinofuranoside, and dimethyl dehydrodiferuloyl diarabinofuranoside), suggesting they are good inhibitors of this enzyme. Based on the chemical composition and evaluated biological activities, the most promising hybrids for generating highly valuable commercial corn hybrids are the white crosses NWP19xNWP81 and NWP27xNWP84, and the yellow cross NYP135xBYP103.

## Figures and Tables

**Table 1:** Seed physical characteristics of high-oil corn hybrids.

Hybrid	Seed Color	HW (kg/hL)	1000-SW (g)	Length (mm)	Width (mm)	Thickness (mm)
NWP11 × NWP84	White	79.22 ± 0.20 ^bcd^	382.26 ± 2.78 ^ab^	13.10 ± 0.99 ^ab^	8.73 ± 0.45 ^bcd^	4.06 ± 0.06 ^cdef^
NWP47 × BWP178	White	81.50 ± 0.27 ^a^	385.97 ± 9.46 ^ab^	12.14 ± 0.63 ^abcd^	7.89 ± 0.29 ^cd^	4.26 ± 0.28 ^cdef^
NWP8 × NWP47	White	81.13 ± 0.58 ^ab^	312.37 ± 4.59 ^f^	12.31 ± 0.64 ^abcd^	8.88 ± 0.52 ^bcd^	4.46 ± 0.15 ^bcd^
NWP81 × NWP19	White	80.82 ± 0.78 ^ab^	280.87 ± 2.26 ^hi^	12.06 ± 0.33 ^abcd^	7.81 ± 0.08 ^d^	3.68 ± 0.29 ^f^
NWP19 × NWP81	White	80.23 ± 0.25 ^abc^	284.01 ± 4.35 ^gh^	11.78 ± 0.19 ^bcd^	8.04 ± 0.25 ^cd^	4.24 ± 0.21 ^cdef^
NWP27 × NWP84	White	81.11 ± 0.89 ^ab^	260.82 ± 10.51 ^i^	11.76 ± 0.35 ^bcd^	7.80 ± 0.48 ^d^	3.80 ± 0.19 ^ef^
NWP13 × NWP85	White	79.91 ± 0.61 ^abc^	397.65 ± 7.44 ^a^	12.69 ± 0.38 ^abcd^	9.12 ± 0.41 ^abc^	4.39 ± 0.02 ^bcde^
NWP32 × NWP9	White	81.16 ± 0.33 ^ab^	304.75 ± 11.70 ^fg^	11.18 ± 0.24 ^d^	8.89 ± 0.37 ^bcd^	5.70 ± 0.25 ^a^
NYP157 × NYP218	Yellow	77.33 ± 0.93 ^de^	368.58 ± 8.82 ^bcde^	11.41 ± 0.19 ^cd^	9.86 ± 0.55 ^ab^	4.64 ± 0.19 ^bc^
CML451/486 × NYP139	Yellow	80.72 ± 0.72 ^abc^	398.22 ± 3.35 ^a^	13.04 ± 0.30 ^abc^	10.38 ± 0.74 ^a^	4.92 ± 0.27 ^b^
NYP135 × BYP103	Yellow	78.53 ± 0.46 ^cde^	379.18 ± 6.88 ^abc^	12.53 ± 0.86 ^abcd^	9.78 ± 0.53 ^ab^	4.47 ± 0.31 ^bcd^
BYP103 × NYP135	Yellow	76.75 ± 0.69 ^ef^	354.66 ± 5.25 ^de^	12.82 ± 0.43 ^abcd^	8.98 ± 0.18 ^bcd^	4.44 ± 0.27 ^bcd^
Armadillo	White	74.55 ± 1.72 ^f^	347.92 ± 0.76 ^e^	12.20 ± 0.83 ^abcd^	8.13 ± 0.48 ^cd^	4.02 ± 0.02 ^cdef^
Garañon	White	66.40 ± 0.80 ^h^	370.77 ± 9.36 ^bcd^	12.18 ± 0.52 ^abcd^	7.94 ± 0.41 ^cd^	3.91 ± 0.03 ^def^
P3140W	White	71.28 ± 0.44 ^g^	358.81 ± 11.02 ^cde^	13.61 ± 0.53 ^a^	7.81 ± 0.12 ^d^	4.42 ± 0.29 ^bcde^

HW: hectoliter weight, 1000-SW: thousand-seed weight. Mean values with different superscript letters in the same column are significantly different (Tukey, *p* ≤ 0.05).

**Table 2:** Extraction yield, phenolic, and flavonoid content of high-oil corn hybrids.

Hybrid	Yield (%)		Total Metabolites	
	Lipid Fraction	Phenolic Fraction	Phenolics (mg GAE/100 g)	Flavonoids (mg CE/100 g)
NWP11 × NWP84	6.50 ± 0.13 ^ab^	0.76 ± 0.06 ^abc^	92.85 ± 4.67 ^cde^	27.21 ± 1.03 ^bcdef^
NWP47 × BWP178	6.08 ± 0.46 ^b^	0.60 ± 0.09 ^bcd^	84.25 ± 5.79 ^ef^	25.57 ± 0.54 ^def^
NWP8 × NWP47	6.32 ± 0.11 ^b^	0.50 ± 0.08 ^cd^	84.56 ± 3.42 ^ef^	24.28 ± 0.95 ^f^
NWP81 × NWP19	6.28 ± 0.65 ^b^	0.43 ± 0.04 ^d^	76.18 ± 3.95 ^f^	24.16 ± 0.37 ^f^
NWP19 × NWP81	7.85 ± 0.91 ^a^	0.81 ± 0.23 ^ab^	130.44 ± 3.08 ^a^	29.97 ± 1.11 ^ab^
NWP27 × NWP84	6.53 ± 0.47 ^ab^	0.65 ± 0.10 ^abcd^	99.12 ± 3.86 ^c^	29.78 ± 0.34 ^abc^
NWP13 × NWP85	5.37 ± 0.42 ^bcd^	0.51 ± 0.02 ^cd^	61.46 ± 2.84 ^g^	26.36 ± 0.89 ^def^
NWP32 × NWP9	5.85 ± 0.57 ^bc^	0.60 ± 0.08 ^bcd^	86.19 ± 2.61 ^def^	27.96 ± 0.55 ^abcde^
NYP157 × NYP218	6.39 ± 0.21 ^b^	0.90 ± 0.06 ^a^	111.91 ± 4.21 ^b^	28.38 ± 1.95 ^abcd^
CML451/486 × NYP139	6.26 ± 0.16 ^b^	0.61 ± 0.02 ^bcd^	98.19 ± 7.29 ^cd^	26.80 ± 0.60 ^cdef^
NYP135 × BYP103	6.57 ± 0.32 ^ab^	0.69 ± 0.04 ^abcd^	102.99 ± 3.11 ^bc^	25.34 ± 1.09 ^def^
BYP103 × NYP135	6.61 ± 0.73 ^ab^	0.72 ± 0.11 ^abc^	100.77 ± 1.28 ^bc^	24.96 ± 1.09 ^ef^
Armadillo	4.69 ± 0.30 ^cde^	0.64 ± 0.08 ^abcd^	79.54 ± 2.86 ^f^	11.64 ± 2.12 ^g^
Garañon	3.88 ± 0.12 ^e^	0.55 ± 0.06 ^bcd^	74.72 ± 3.02 ^f^	30.81 ± 0.39 ^a^
P3140W	4.39 ± 0.37 ^de^	0.52 ± 0.09 ^cd^	81.20 ± 5.19 ^ef^	28.23 ± 0.56 ^abcd^

Values are the mean ± standard deviation (three replicates), and they are expressed on a dry weight basis. GAE, gallic acid equivalents; CE, catechin equivalents. Means with different superscript letters in the same column are significantly different (Tukey, *p* ≤ 0.05).

**Table 3:** Compounds identified by UPLC-DAD-MS in the phenolic fraction of mature seeds from high-oil corn hybrids.

Peak	RT (min)	Experimental *m*/*z* [M−H]^−^	Main Fragments (Ion Intensity)	Proposed Compound	Reference
1	22.74	366	160 (85)	Tyrosyl-tryptophan (−) (TT)	[15]
2	24.73	595	545 (10), 367 (55), 317 (20), 193 (25)	Dehydrotriferulic acid, hydrated (TFA, hydrated)	[15]
3	25.70	677	531 (27), 339 (40), 193 (85)	Dimethyl dehydrodiferuloyl diarabinofuranoside I (DFA-MeAra_2_ I)	[16]
4	26.12	339	193 (20), 175 (100)	Methyl 5-*O*-feruloyl arabinofuranoside I (FA-MeAra I)	[16]
5	26.63	339	193 (30), 175 (100)	Methyl 5-*O*-feruloyl arabinofuranoside II (FA-MeAra II)	[16]
6	27.22	663	517 (37), 339 (20), 325 (13), 193 (20), 175 (30)	Dehydrodiferuloyl diarabinofuranoside (DFA-Ara_2_)	[16]
7	27.99	677	531(25), 339 (45), 193 (20)	Dimethyl dehydrodiferuloyl diarabinofuranoside II (DFA-MeAra_2_ II)	[16]
8	28.53	677	531 (40), 339 (20), 193 (25)	Dimethyl dehydrodiferuloyl diarabinofuranoside III (DFA-MeAra_2_ III)	[16]
9	29.22	439	289 (100), 274 (5), 149 (60)	*N,N’*-Diferuloyl putrescine I (DFP I)	[15,18]
10	29.97	677	531 (35), 339 (20), 193 (25)	Dimethyl dehydrodiferuloyl diarabinofuranoside IV (DFA-MeAra_2_ IV)	[16]
11	30.61	677	531 (50), 339 (25), 193 (20)	Dimethyl dehydrodiferuloyl diarabinofuranoside V (DFA-MeAra_2_ V)	[16]
12	31.26	409	233 (20), 175 (40)	*p-*Coumaroyl feruloyl putrescine (*p-*CFP)	[15,17]
13	31.76	439	248 (54), 175 (35)	*N,N’*-Diferuloyl putrescine II (DFP II)	[15,18]
14	32.19	677	531 (15), 339 (60), 193 (40)	Dimethyl dehydrodiferuloyl diarabinofuranoside VI (DFA-MeAra2 VI)	[16]
15	36.06	877	439 (40)	*bis-N,N’*-Diferuloyl putrescine I (*bis*-DFP I)	[15]
16	37.28	877	439 (45)	*bis-N,N’*-Diferuloyl putrescine II (*bis*-DFP II)	[15]
17	40.39	877	439 (30)	*bis-N,N’*-Diferuloyl putrescine III (*bis*-DFP III)	[15]
18	41.48	877	439 (20)	*bis-N,N’*-Diferuloyl putrescine IV (*bis*-DFP IV)	[15]
19	41.98	877	439 (20)	*bis-N,N’*-Diferuloyl putrescine V (*bis*-DFP V)	[15]

**Table 4:** Antioxidant capacity of the phenolic fraction of high-oil corn hybrids.

Hybrid	Antioxidant Capacity (µmol TE/100 g)	
	DPPH	ABTS
NWP11 × NWP84	538.92 ± 38.59 ^cde^	1797.54 ± 47.50 ^bcde^
NWP47 × BWP178	589.31 ± 62.91 ^bcd^	1844.10 ± 139.90 ^bcde^
NWP8 × NWP47	469.56 ± 25.04 ^de^	1578.59 ± 80.52 ^d^
NWP81 × NWP19	564.09 ± 59.56 ^bcd^	1777.00 ± 299.08 ^cde^
NWP19 × NWP81	780.39 ± 42.39 ^a^	2498.58 ± 214.14 ^a^
NWP27 × NWP84	643.17 ± 76.64 ^abc^	2037.52 ± 40.94 ^bcd^
NWP13 × NWP85	383.04 ± 22.96 ^e^	1135.26 ± 32.63 ^e^
NWP32 × NWP9	567.67 ± 50.52 ^bcd^	1925.85 ± 34.98 ^bcde^
NYP157 × NYP218	716.06 ± 81.15 ^ab^	2218.40 ± 212.81 ^ab^
CML451/486 × NYP139	590.91 ± 31.24 ^bcd^	1924.74 ± 83.26 ^bcde^
NYP135 × BYP103	641.70 ± 40.66 ^abc^	2155.48 ± 107.39 ^abc^
BYP103 × NYP135	585.66 ± 62.63 ^bcd^	2098.66 ± 205.84 ^abcd^
Armadillo	511.01 ± 23.71 ^cde^	1677.03 ± 98.92 ^cd^
Garañon	593.72 ± 25.55 ^bcd^	1841.78 ± 116.06 ^bcde^
P3140W	608.44 ± 80.81 ^bcd^	2082.19 ± 159.96 ^abcd^

Values are the mean ± standard deviation (three replicates), and they are expressed on a dry weight basis. TE, Trolox equivalents. Means with different superscript letters in the same column are significantly different (Tukey, *p* < 0.05).

**Table 5:** Molecular docking binding energies of α-glucosidase enzyme with selected phenolic compounds identified in HOC extracts and acarbose.

Compound	Binding Energy (kcal/mol)
Dehydrodiferuloyl diarabinofuranoside (DFA Ara_2_)	−7.78
Dimethyl dehydrodiferuloyl diarabinofuranoside III (DFA-MeAra_2_ III)	−7.25
*N,N’*-Diferuloyl putrescine (DFP)	−6.52
*p*-Coumaroyl-feruloyl putrescine (*p*-CFP)	−6.30
Acarbose	−6.50

## Data Availability

The original contributions presented in this study are included in the article/Supplementary Materials. Further inquiries can be directed to the corresponding author.

## Supplementary Materials

The following supporting information can be downloaded at: https://www.mdpi.com/article/10.3390/molecules31101654/s1, Table S1: Linear correlation coefficients between physical characteristics, phenolic content, antioxidant capacity and alfa-glucosidase inhibition of high-oil corn; Table S2: Content of phenolic compounds (mg/100 g d.w.) identified by UPLC-DAD-MS in the methanol extracts of mature seeds from high-oil corn hybrids; Figure S1: Molecular docking and interactions of the DFA-ARA_2_ compound and the reference drug acarbose in the active site of α-glucosidase. Figure S2: Cluster analysis of network correlation based on phenolics, antioxidant capacity and α-glucosidase inhibition of high-oil corn hybrids.

## Abbreviations

The following abbreviations are used in this manuscript:

HOC	High-oil corn
AC	Antioxidant capacity
αGI	α-glucosidase inhibitory activity
ABTS	2,2’-Azino-bis(3-ethylbenzothiazoline-6-sulfonic acid)
DPPH	2,2-diphenyl-1-picrylhydrazyl
DH	Double haploid
NWP	Northwest White Population
BWP	Bajío White Population
NYP	Northwest Yellow Population
BYP	Bajío Yellow Population
HW	Hectoliter weight
1000-SW	Thousand-seed weight
TP	Total phenolics
TF	Total flavonoids
GAE	Gallic acid equivalents
CE	Catechin equivalents
UPLC	Ultra-performance liquid chromatography
DAD	Diode array detector
MS	Mass spectrometry
PCA	Principal component analysis
