# Supplementary material for: Phenolic Profile, Antioxidant Capacity, and Alpha-Glucosidase Inhibitory Activity of High-Oil Corn Doubled-Haploid Hybrids from Mexico
Source: Molecules. 2026 May 14;31(10):1654. doi: 10.3390/molecules31101654 (PMC13209748; doi:10.3390/molecules31101654)
Supplement: Supplementary file 1 [file molecules-31-01654-s001.zip › Suppl. Figure 1.pdf]

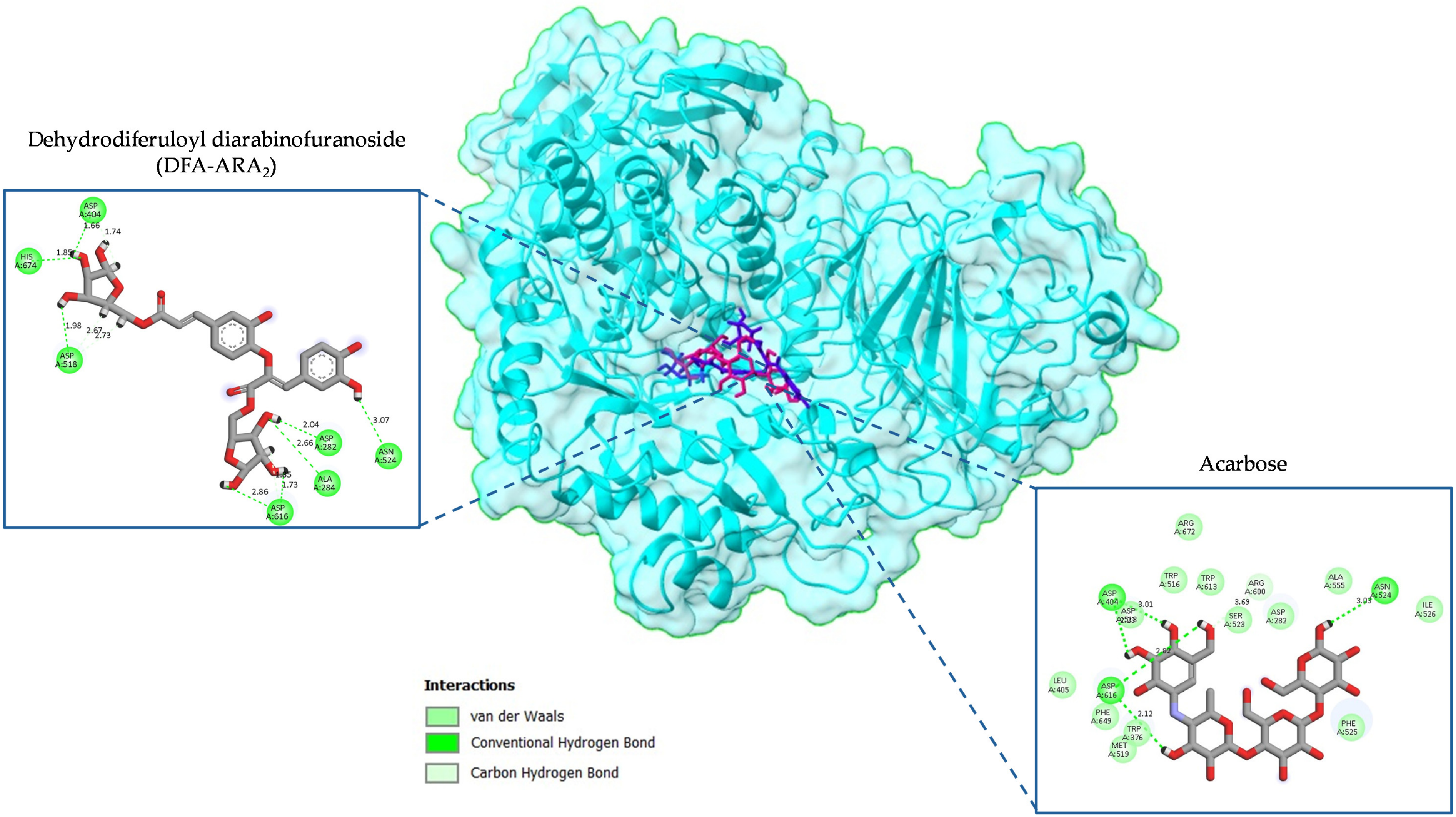

Supplementary Figure S1. Molecular docking and interactions of the DFA-ARA2 compound and the reference drug acarbose in the active site of α-glucosidase.
