# Supplementary material for: Phenolic Profile, Antioxidant Capacity, and Alpha-Glucosidase Inhibitory Activity of High-Oil Corn Doubled-Haploid Hybrids from Mexico
Source: Molecules. 2026 May 14;31(10):1654. doi: 10.3390/molecules31101654 (PMC13209748; doi:10.3390/molecules31101654)
Supplement: Supplementary file 1 [file molecules-31-01654-s001.zip › Suppl. Figure 2.pdf]

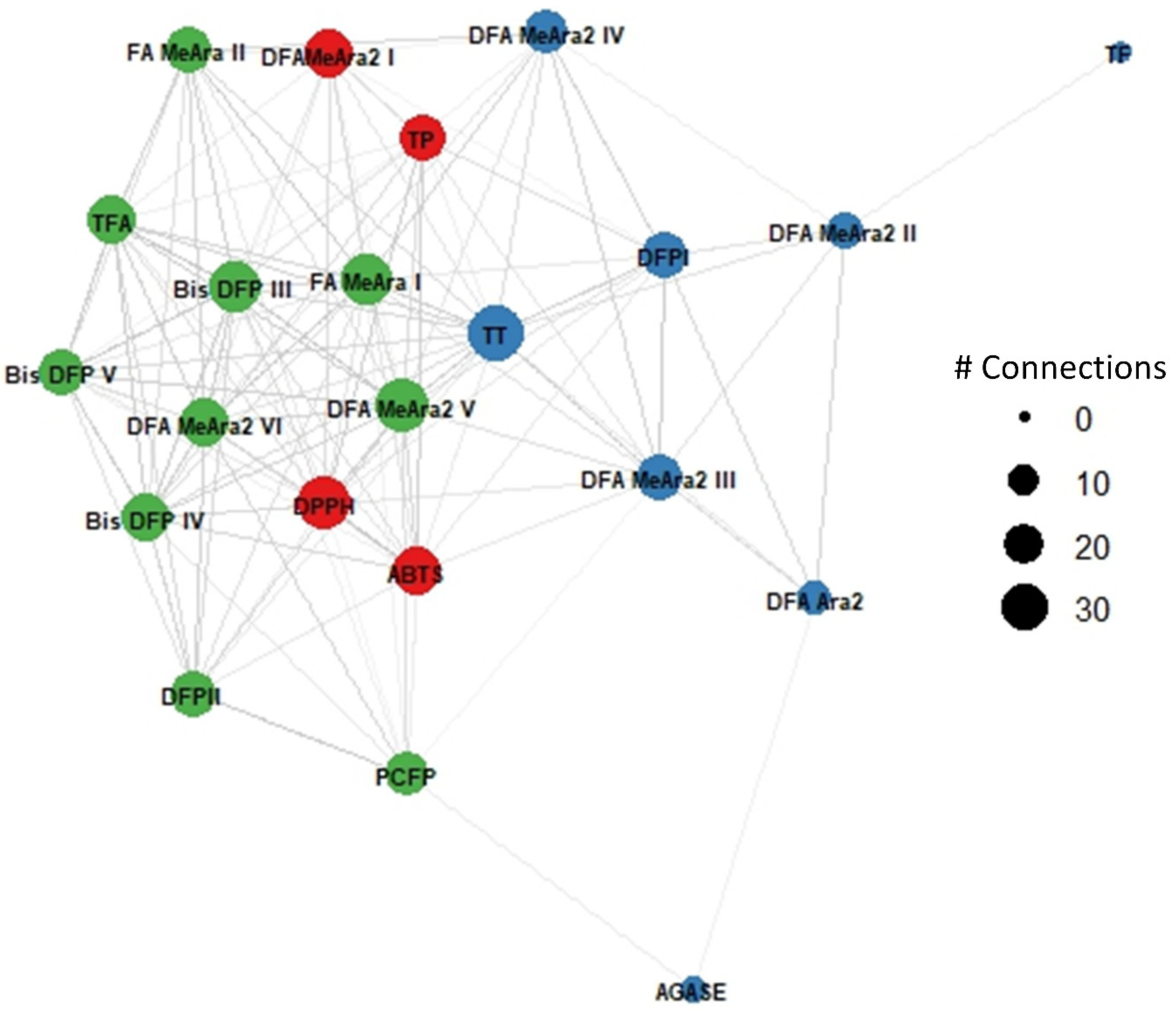

Supplementary Figure S2. Cluster analysis of network correlation based on phenolics, antioxidant capacity and α-glucosidase inhibition of high-oil corn hybrids. The nodes represent the analyzed variables, and the edges (lines) indicate the statistical correlations between them. The color of the nodes identifies clusters (Cluster 1: red; Cluster 2: blue; Cluster 3: green) grouping variables with similar behavior. The size of the nodes indicates the strength of the connections within the network (range 0-30). Positive correlations and $r \geq 0.4$ values were considered for the analysis. TT: Tyrosil-tryptophan; TFA: Dehydrotriferulic acid, hydrated; DFA-MeAra2: Dimethyl dehydro-diferuloyl diarabinofuranoside I, II, III, IV, V, VI; Fa-MeAra: Methyl 5-O-feruloyl arabinofuranoside I, II; DFA-Ara2: Dehydrodiferuloyl diarabinofuranoside; *p*-CFP: p-Coumaroyl-feruloyl putrescine; DFP: N,N′-Diferuloyl putrescine I, II; Bis-DFP: bis-N,N′-Diferuloyl putrescine III, IV, V; AGASE: α-glucosidase inhibition; TP, Total phenolics; TF, Total flavonoids; ABTS and DPPH, antioxidant capacity methods.
