# Supplementary material for: Phenolic Profile, Antioxidant Capacity, and Alpha-Glucosidase Inhibitory Activity of High-Oil Corn Doubled-Haploid Hybrids from Mexico
Source: Molecules. 2026 May 14;31(10):1654. doi: 10.3390/molecules31101654 (PMC13209748; doi:10.3390/molecules31101654)
Supplement: Supplementary file 1 [file molecules-31-01654-s001.zip › Suppl. Table 1.pdf]

Supplementary Table S1. Linear correlation coefficients between physical characteristics, phenolics content, antioxidant capacity and alpha-glucosidase inhibition of high-oil corn.

Variables	HW	1000-SW	L	W	T	Phenolic fraction (%)	Lipid fraction (%)	TP	TF	DPPH	ABTS	α-Glucosidase inhibition	TT	TFA	DFA MeAra2 I	FA MeAra I	FA MeAra II	DFA Ara2	DFA MeAra2 II	DFA MeAra2 III	pCFP	DFA MeAra2 IV	DFA MeAra2 V	NN CFP	DFP	DFA MeAra2 VI	Bis DFP I	Bis DFP II	Bis DFP III	Bis DFP IV	Bis DFP V
**HW**	1																														
**1000-SW**	-0.29																														
**L**	-0.22	0.44																													
**W**	0.24	0.46	0.14																												
**T**	0.23	0.16	-0.12	0.48																											
**Phenolic fraction (%)**	0.07	0.05	-0.17	0.28	0.07	1																									
**Lipid fraction (%)**	0.72	-0.32	-0.15	0.23	0.08	0.36	1																								
**TP**	0.25	-0.26	-0.18	0.21	0.13	0.69	0.69	1																							
**TF**	-0.02	-0.08	-0.09	0.01	0.12	0.15	0.17	0.27	1																						
**DPPH**	-0.05	-0.27	-0.28	-0.05	0.03	0.53	0.40	0.77	0.37	1																					
**ABTS**	-0.08	-0.27	-0.18	-0.01	0.09	0.54	0.35	0.78	0.35	0.87	1																				
**α- Glucosidase inh.**	0.07	-0.14	-0.04	0.05	-0.05	0.11	0.30	0.25	0.01	0.19	0.31	1																			
**TT**	0.26	-0.56	-0.19	-0.24	-0.29	0.04	0.43	0.44	0.21	0.53	0.47	0.20	1																		
**TFA**	-0.1	-0.31	-0.06	-0.21	-0.33	0.22	0.31	0.45	0.23	0.57	0.51	0.06	0.70	1																	
**DFA-MeAra2 I**	0.09	-0.09	-0.23	0.23	0.03	0.25	0.28	0.55	0.25	0.60	0.53	-0.13	0.52	0.45	1																
**FA-MeAra I**	-0.28	-0.38	-0.28	-0.37	-0.43	0.05	-0.03	0.23	0.19	0.53	0.37	0.04	0.74	0.67	0.52	1															
**FA-MeAra II**	-0.17	-0.4	-0.41	-0.29	-0.36	0.16	0.04	0.23	0.18	0.50	0.34	-0.08	0.61	0.69	0.48	0.84	1														
**DFA-Ara2**	0.42	-0.62	-0.17	-0.02	-0.04	-0.09	0.47	0.26	0.18	0.07	0.12	0.43	0.49	-0.04	0.03	0.15	-0.05	1													
**DFA-MeAra2 II**	0.37	-0.34	0.12	-0.11	-0.35	-0.08	0.46	0.16	0.47	0.07	0.04	0.23	0.55	0.19	0.06	0.22	0.10	0.68	1												
**DFA-MeAra2 III**	0.16	-0.46	-0.21	-0.19	-0.13	0.27	0.35	0.51	0.30	0.52	0.50	0.31	0.70	0.28	0.35	0.54	0.33	0.58	0.53	1											
**DFP I**	0.24	-0.44	-0.16	-0.04	-0.05	0.27	0.43	0.57	0.26	0.49	0.49	0.32	0.71	0.27	0.41	0.49	0.27	0.64	0.55	0.97	1										
**DFA-MeAra2 IV**	-0.02	-0.4	-0.2	-0.17	-0.46	0.22	0.15	0.34	0.16	0.40	0.30	0.03	0.59	0.31	0.42	0.69	0.65	0.34	0.45	0.70	0.69	1									
**DFA-MeAra2 V**	-0.09	-0.29	0.03	-0.11	-0.3	0.34	0.37	0.61	0.36	0.68	0.68	0.25	0.73	0.78	0.42	0.57	0.53	0.23	0.39	0.56	0.55	0.51	1								
**p-CFP**	-0.46	0.09	0.12	-0.02	-0.07	0.05	-0.08	0.23	0.25	0.41	0.47	0.47	0.39	0.40	0.23	0.43	0.17	0.19	0.09	0.41	0.37	0.10	0.59	1							
**DFP II**	-0.46	-0.04	0.07	-0.2	-0.17	0.02	-0.06	0.26	0.20	0.47	0.49	0.32	0.50	0.63	0.30	0.56	0.31	0.09	0.04	0.32	0.27	0.05	0.63	0.91	1						
**DFA-MeAra2 VI**	-0.57	-0.17	-0.1	-0.17	-0.33	0.21	-0.11	0.35	0.26	0.58	0.54	0.09	0.53	0.72	0.48	0.80	0.73	0.01	0.06	0.40	0.36	0.51	0.68	0.66	0.75	1					
**bis-DFP I**	0.04	-0.24	-0.47	0.03	0.66	0.04	-0.07	0.01	0.16	0.10	0.14	-0.03	-0.11	-0.09	0.02	-0.09	0.09	-0.02	-0.34	0.06	0.02	-0.22	-0.19	-0.04	-0.07	-0.04	1				
**bis-DFP II**	0.05	0.27	0.24	0.15	-0.17	-0.06	-0.06	-0.10	-0.29	-0.05	-0.08	-0.36	-0.07	0.16	0.21	-0.10	0.14	-0.54	-0.25	-0.56	-0.50	-0.09	0.04	-0.33	-0.20	-0.09	-0.38	1			
**bis-DFP III**	-0.24	-0.36	-0.2	-0.26	-0.39	0.33	0.20	0.49	0.16	0.62	0.59	0.09	0.65	0.89	0.44	0.70	0.75	0.04	0.12	0.32	0.27	0.43	0.80	0.44	0.64	0.81	-0.10	0.14	1		
**bis-DFP IV**	-0.1	-0.41	-0.34	-0.2	-0.1	0.34	0.34	0.60	0.19	0.67	0.64	0.19	0.61	0.82	0.42	0.61	0.57	0.15	-0.01	0.40	0.35	0.21	0.68	0.51	0.71	0.68	0.16	-0.19	0.85	1	
**bis-DFP V**	-0.06	-0.35	-0.09	-0.15	-0.26	0.16	0.26	0.42	0.06	0.49	0.45	-0.04	0.61	0.84	0.35	0.49	0.57	0.05	0.04	0.18	0.16	0.25	0.78	0.37	0.56	0.58	-0.11	0.28	0.85	0.77	1

Significant positive correlation at $p \le 0.05$ $p \le 0.01$ $p \le 0.001$

Significant negative correlation at $p \le 0.05$ $p \le 0.01$ $p \le 0.001$
