# Supplementary material for: Phenolic Profile, Antioxidant Capacity, and Alpha-Glucosidase Inhibitory Activity of High-Oil Corn Doubled-Haploid Hybrids from Mexico
Source: Molecules. 2026 May 14;31(10):1654. doi: 10.3390/molecules31101654 (PMC13209748; doi:10.3390/molecules31101654)
Supplement: Supplementary file 1 [file molecules-31-01654-s001.zip › Suppl. Table 2.pdf]

Supplementary Table S2. Content of phenolic compounds (mg/100 g d.w.) identified by UPLC-DAD-MS in the methanol extracts of mature seeds from high-oil corn hybrids.

Genotype / Compound	NWP11 x NWP84	NWP47 x BWP178	NWP8 x NWP47	NWP81 x NWP19	NWP19 x NWP81	NWP27 x NWP84	NWP13 x NWP85	NWP32 x NWP9	NYP157 x NYP218	CML451/486 x NYP139	NYP135 x BYP103	BYP103 x NYP135	Armadillo	Garañon	P3140W
TT	157.55 ±10.11^{def}	156.50±16.65^{def}	145.74±1.66^{ef}	273.48±3.64^{ab}	285.38±5.51^{a}	242.79±5.79^{c}	114.91±7.61^{gh}	147.09±5.97^{ef}	152.95±5.79^{def}	169.16±14.67^{de}	253.09±3.59^{bc}	94.36±1.81^{h}	139.73±12.97^{fg}	145.41±14.92^{ef}	176.18±6.20^{d}
TFA	4.70±0.51^{bcd}	3.18±0.35^{e}	2.58±0.22^{e}	7.48±1.21^{a}	8.33±0.11^{a}	3.32±0.47^{e}	2.40±0.01^{e}	2.83±0.01^{e}	5.76±0.03^{b}	3.56±0.40^{cde}	4.98±0.12^{b}	3.08±0.23^{e}	3.47±0.30^{de}	4.79±0.24^{bc}	5.15±0.64^{b}
DFA-Ara$_2$	1.61±0.19^{gh}	0.81±0.02^{i}	4.71±0.31^{ab}	3.06±0.17^{d}	3.96±0.20^{c}	5.28±0.38^{a}	2.41±0.26^{ef}	3.05±0.17^{d}	0.63±0.06^{i}	2.04±0.02^{fg}	4.49±0.29^{bc}	2.93±0.28^{de}	1.17±0.14^{hi}	1.68±0.13^{gh}	1.10±0.02^{hi}
DFA-MeAra$_2$ I	23.22±0.01^{fgh}	29.76±4.45^{bcdef}	23.28±2.96^{fgh}	29.39±3.60^{cdef}	36.49±0.43^{ab}	29.97±0.78^{bcdef}	16.76±1.16^{h}	26.04±1.92^{defg}	33.68±3.09^{bc}	40.72±0.84^{a}	31.50±0.36^{bcd}	19.33±0.73^{gh}	24.81±0.47^{defg}	30.24±4.08^{bcde}	23.91±0.72^{efg}
DFA-MeAra$_2$ II	4.08±0.08^{b}	1.95±0.18^{gh}	2.93±0.23^{de}	3.67±0.33^{bc}	3.56±0.23^{bc}	4.95±0.04^{a}	3.13±0.11^{cd}	1.59±0.09^{h}	0.98±0.11^{i}	2.56±0.20^{ef}	3.53±0.32^{bc}	2.35±0.24^{fg}	0.14±0.01^{j}	2.36±0.06^{fg}	1.87±0.18^{gh}
DFA-MeAra$_2$ III	4.90±0.10^{d}	3.33±0.07^{f}	1.04±0.14^{jk}	2.04±0.30^{hi}	12.00±0.54^{b}	14.76±0.65^{a}	1.19±0.01^{ijk}	5.83±0.40^{c}	1.88±0.20^{hij}	2.39±0.11^{gh}	11.98±0.06^{b}	1.00±0.06^{k}	3.23±0.12^{fg}	3.75±0.35^{ef}	4.54±0.16^{de}
DFA-MeAra$_2$ IV	3.25±0.07^{bc}	1.95±0.15^{e}	2.07±0.01^{e}	3.28±0.12^{bc}	3.67±0.25^{b}	6.24±0.04^{a}	2.19±0.08^{de}	1.88±0.17^{e}	3.64±0.06^{b}	3.11±0.10^{c}	3.67±0.09^{b}	1.72±0.12^{e}	3.45±0.36^{bc}	3.45±0.18^{bc}	2.62±0.22^{d}
DFA-MeAra$_2$ V	3.97±0.43^{de}	2.59±0.01^{fg}	2.67±0.05^{fg}	4.41±0.32^{bcd}	5.42±0.16^{a}	4.20±0.02^{cd}	1.74±0.15^{h}	2.17±0.27^{gh}	4.44±0.42^{bcd}	2.84±0.21^{fg}	5.09±0.33^{ab}	3.12±0.02^{f}	2.29±0.30^{gh}	3.24±0.16^{ef}	4.79±0.17^{abc}
DFA-MeAra$_2$ VI	1.91±0.20^{fg}	0.81±0.03^{i}	1.07±0.07^{hi}	2.92±0.09^{cd}	3.92±0.02^{b}	2.36±0.05^{ef}	0.77±0.04^{i}	1.35±0.14^{h}	3.07±0.30^{cd}	2.02±0.22^{f}	3.42±0.16^{bc}	1.49±0.11^{gh}	2.37±0.02^{ef}	4.58±0.29^{a}	2.88±0.35^{de}
Fa-MeAra I	19.38±0.53^{ghi}	22.52±2.70^{efg}	17.51±1.01^{hi}	33.38±0.27^{ab}	35.89±0.82^{a}	32.90±0.12^{ab}	21.65±0.25^{fg}	20.20±0.65^{fgh}	27.74±1.62^{c}	23.58±2.92^{def}	29.45±0.30^{bc}	15.88±0.99^{i}	27.07±1.28^{cd}	34.82±1.16^{a}	25.89±1.73^{cde}
Fa-MeAra II	32.67±1.48^{de}	29.59±1.52^{ef}	23.15±1.15^{g}	49.14±3.93^{a}	41.79±1.22^{bc}	42.37±2.10^{b}	25.62±1.77^{fg}	31.85±1.28^{def}	46.06±3.71^{ab}	31.22±0.32^{def}	33.90±0.67^{de}	22.89±2.72^{g}	35.92±0.41^{cd}	43.93±3.20^{ab}	31.76±1.43^{def}
DFP I	4.55±0.30^{d}	0.49±0.06^{g}	0.48±0.05^{g}	1.53±0.03^{f}	15.11±0.56^{b}	17.82±0.21^{a}	0.69±0.01^{fg}	6.20±0.59^{c}	0.66±0.06^{fg}	5.70±0.60^{c}	15.19±0.39^{b}	0.81±0.01^{fg}	3.00±0.06^{e}	1.49±0.22^{f}	3.84±0.20^{de}
p-CFP	2.44±0.28^{i}	4.23±0.27^{ef}	3.50±0.32^{fgh}	4.81±0.15^{e}	7.90±0.17^{c}	3.37±0.49^{gh}	2.21±0.04^{i}	3.74±0.26^{fg}	4.25±0.24^{ef}	3.30±0.12^{gh}	14.61±0.01^{a}	5.78±0.01^{d}	2.82±0.13^{hi}	9.93±0.30^{b}	9.24±0.41^{b}
DFP II	6.29±0.24^{i}	15.46±0.08^{f}	11.40±1.36^{g}	22.59±1.57^{e}	35.73±0.31^{b}	3.94±0.04^{j}	4.07±0.20^{j}	8.83±0.19^{h}	12.55±0.05^{g}	8.50±0.46^{hi}	39.00±0.96^{a}	14.90±0.35^{f}	11.16±0.62^{g}	33.28±0.43^{c}	30.45±1.29^{d}
Bis-DFP I	3.34±0.19^{def}	4.62±0.16^{def}	2.83±0.13^{ef}	5.17±0.53^{de}	6.14±0.44^{cd}	3.97±0.12^{def}	2.07±0.11^{f}	36.00±3.53^{a}	10.74±0.86^{b}	2.62±0.30^{ef}	4.73±0.32^{def}	4.03±0.44^{def}	5.53±0.25^{de}	8.81±0.20^{bc}	5.46±0.70^{de}
Bis-DFP II	40.72±4.39^{b}	41.72±1.87^{b}	31.73±1.24^{c}	58.66±3.39^{a}	12.98±0.04^{de}	16.00±0.08^{d}	19.95±0.91^{d}	6.44±0.33^{e}	53.09±1.99^{a}	58.97±4.20^{a}	14.74±0.97^{d}	28.37±0.26^{c}	40.81±1.01^{b}	17.29±0.99^{d}	41.87±4.39^{b}
Bis-DFP III	3.12±0.31^{def}	1.92±0.23^{hi}	2.19±0.31^{ghi}	4.73±0.35^{ab}	5.27±0.37^{a}	2.69±0.28^{fgh}	0.90±0.02^{j}	1.66±0.10^{ij}	4.45±0.44^{bc}	1.82±0.09^{i}	3.59±0.15^{de}	2.25±0.20^{ghi}	2.86±0.25^{efg}	3.71±0.09^{cd}	3.24±0.28^{def}
Bis-DFP IV	1.87±0.05^{f}	2.36±0.12^{ef}	2.39±0.27^{ef}	3.95±0.40^{bc}	6.89±0.26^{a}	1.94±0.27^{f}	0.80±0.01^{g}	2.84±0.25^{de}	4.48±0.40^{b}	0.97±0.01^{g}	4.09±0.39^{bc}	2.19±0.14^{ef}	2.56±0.10^{ef}	3.42±0.07^{cd}	2.79±0.14^{de}
Bis-DFP V	2.96±0.08^{de}	2.39±0.14^{ef}	3.42±0.27^{cd}	4.54±0.41^{a}	4.55±0.09^{a}	2.56±0.17^{ef}	1.75±0.11^{g}	2.20±0.15^{fg}	4.39±0.17^{a}	2.30±0.18^{fg}	3.62±0.09^{bc}	2.19±0.14^{fg}	2.95±0.30^{de}	2.67±0.28^{ef}	4.06±0.08^{ab}
Total	322.53	326.17	284.68	518.22	534.98	441.42	225.23	311.79	375.44	367.39	484.68	228.67	315.31	358.86	381.64

Values are the mean ± standard deviation (three replicates) and they are expressed on a dry weight basis (mg/100 g d.w.). LOD: ferulic acid (5.48 µg/mL), tryptophan (128.95 µg/mL). TT: Tyrosil-tryptophan; TFA: Dehydrotriferulic acid, hydrated; DFA-MeAra$_2$: Dimethyl dehydrodiferuloyl diarabinofuranoside I, II, III, IV, V, VI; Fa-MeAra: Methyl 5-O-feruloyl arabinofuranoside I, II; DFA-Ara$_2$: Dehydrodiferuloyl diarabinofuranoside; *p*-CFP: *p*-Coumaroyl-feruloyl putrescine; DFP: *N,N'*-Diferuloyl putrescine; *Bis*-DFP: *bis-N,N'*-Diferuloyl putrescine I, II, III, IV, V. Different superscript letters in a single row indicate significant differences between the genotypes (Tukey, α=0.05).
